# ENet-CAEM: a field strawberry disease identification model based on improved EfficientNetB0 and multiscale attention mechanism

**DOI:** 10.3389/fpls.2025.1701740

**Published:** 2025-12-01

**Authors:** Jiajiao Chang, Honghui Li, Xueliang Fu, Yuanyuan Jiao

**Affiliations:** 1 College of Computer and Information Engineering, Inner Mongolia Agricultural University, Hohhot, China; 2 Key Laboratory of Smart Animal Husbandry at Universities of Inner Mongolia Autonomous Region, Hohhot, China

**Keywords:** strawberry disease classification, EfficientNetB0, deep learning, multi-scale featurefusion, image classification

## Abstract

**Introduction:**

Real-time diagnosis of strawberry diseases plays a key role in sustaining yield and improving field management. However, achieving reliable recognition remains challenging. Lesions often display irregular shapes and appear at different scales, which complicates detection. Field images also contain cluttered backgrounds, while many diseases look visually alike, making differentiation more difficult. In addition, collecting data under real conditions is not easy, resulting in small datasets on which deep learning models tend to overfit and fail to generalize.

**Methods:**

To address these issues, this study introduces ENet-CAEM, a redesigned EfficientNetB0 framework equipped with modules tailored for disease recognition. The Channel Context Module helps the network capture key lesion features while suppressing background noise. The Multi-Scale Efficient Channel Attention module applies multiple one-dimensional filters of varying sizes in parallel, enabling the model to highlight critical patterns, tell apart similar diseases, and adapt to lesions of different scales. A lightweight version of Atrous Spatial Pyramid Pooling is further integrated, allowing the network to perceive features at multiple spatial ranges. To balance local detail with global context, a mixed pooling strategy is adopted, enhancing robustness when lesion shapes change. Finally, Learnable DropPath and label smoothing are applied as regularization strategies, reducing overfitting and improving generalization on limited data.

**Results:**

Experiments show that ENet-CAEM achieves 85.84% accuracy on a self-built dataset, outperforming the baseline by 4.29%. On a public strawberry dataset, the model reaches 97.39%, surpassing existing approaches.

**Discussion:**

The proposed ENet-CAEM model shows superior accuracy and robustness over existing methods, providing an effective solution for strawberry disease recognition in practical field environments.

## 1 Introduction

China is the world’s largest strawberry producer and has the greatest cultivation area (Liu et al., 2023). Strawberries have strong economic and nutritional value, containing bioactive nutrients linked to lower risks of cancer, high cholesterol, and heart disease (Liu et al., 2020a). However, diseases are common during the leaf and fruit stages, and inexperienced farmers often find it difficult to identify them quickly and apply appropriate control measures. Delays reduce quality and yield and cause major economic losses. (Barbedo, 2022) pointed out that traditional manual diagnosis is slow, labor-intensive, and subjective, making early and accurate disease diagnosis difficult. Thus, an efficient and accurate strawberry disease identification model is essential for timely detection, reliable diagnosis, and precise management.

With advances in artificial intelligence, machine learning has become essential for intelligent recognition, enabling computers to learn from data (Bishop and Nasrabadi, 2006). Deep learning, using multilayer neural networks, improves hierarchical feature representation and generalization (Hinton and Salakhutdinov, 2006). Convolutional neural networks demonstrated success in visual recognition, such as handwritten digit classification (LeCun et al., 2002), and AlexNet (Krizhevsky et al., 2012) achieved breakthroughs on ImageNet, popularizing deep convolutional networks. Later models such as ZFNet (Zeiler and Fergus, 2014), VGGNet (Simonyan and Zisserman, 2014), and GoogLeNet (Szegedy et al., 2015) further enhanced performance through architectural optimization and multi-scale feature fusion. These advances have extended deep learning to applications like agricultural image analysis.

Recent studies have made significant progress in plant disease classification. (Ramdani and Suyanto, 2021) utilized convolutional neural networks (CNNs) to detect strawberry leaf images, comparing VGG16, ResNet50, and G-Net, with ResNet50 achieving the highest accuracy. (Hassan et al., 2021) classified 38 disease categories across 14 plants in the PlantVillage dataset using InceptionV3, InceptionResNetV2, MobileNetV2, and EfficientNetB0, achieving high accuracy. (Degadwala et al., 2023) proposed a hop disease classification method based on transfer learning, comparing AlexNet, VGG16, and ResNet50. However, most of these approaches rely on “heavyweight” architectures with large parameter counts and high computational demands, limiting their deployment in resource-constrained agricultural scenarios.

In practical applications, disease recognition systems are often deployed on mobile devices, edge platforms, or low-power monitoring systems with limited computing resources, which cannot support the high demands of complex deep neural networks. Therefore, developing a lightweight strawberry disease recognition model that maintains accuracy while reducing computational cost and ensuring fast response is crucial for real-world agricultural deployment.

To enhance recognition efficiency and deployment adaptability, some studies focus on lightweight model design and architectural improvements. (Wang and Cui, 2024) proposed a strawberry disease recognition method based on MobileNetV3-Small, using data augmentation, an enhanced Inception_ A module, the ULSAM attention mechanism, and CondConv replacement to achieve high accuracy with fewer parameters. (Hossain et al., 2018) developed a lightweight CNN framework with a fine-tuned VGG16, achieving high fruit classification accuracy on two datasets. (Tian et al., 2019) introduced a five-layer CNN for corn disease recognition, obtaining relatively high accuracy. However, in natural environments, factors such as noise and varying light conditions pose additional challenges for image feature extraction and recognition.

In further research, (Wu et al., 2025) proposed D-YOLO, a lightweight model for strawberry health recognition that achieves an effective balance between accuracy and detection speed. (He et al., 2024) developed KTD-YOLOv8, based on YOLOv8, integrating KernelWarehouse convolution, the Triplet Attention mechanism, and a DBB parameter-sharing structure to enhance strawberry leaf disease detection. However, recognition robustness and multi-scale adaptability remain limited in complex environments with overlapping or unevenly distributed lesions. (Xu et al., 2021) introduced TCI-ALEXN, an enhanced AlexNet incorporating Inception modules, global pooling, and transfer learning, which effectively identified four types of corn diseases but still exhibited confusion among visually similar categories. (Wongsila et al., 2021) designed a CNN-based system for detecting mango anthracnose, achieving over 70% accuracy, though its performance was constrained by limited training data and low classification precision.

In summary, existing methods for strawberry disease identification still face several challenges. First, the high visual similarity between complex backgrounds and disease features in natural environments often leads to misclassification. Second, the irregular morphology and significant scale variation of lesions restrict the effectiveness of traditional models in multi-scale feature extraction. Third, limited sample availability and class imbalance weaken the model’s generalization capability, reducing its practicality in real-world agricultural applications.

Given the above limitations, this paper proposes a lightweight strawberry disease recognition framework, ENet-CAEM, which integrates multiple structural optimizations to enhance multi-scale lesion detection, background suppression, and small-sample learning. The main contributions are as follows: (1) A Channel Context Module (CCM) is introduced to reduce background interference through channel-level context modeling. Additionally, a Multi-Scale Efficient Channel Attention (MultiScaleECA) module with lightweight spatial attention strengthens the model’s focus on lesion textures and edges while suppressing background noise. (2) A Lightweight Atrous Spatial Pyramid Pooling (LightASPP) module expands the receptive field using different atrous rates and incorporates a Mixed Pooling (MP) strategy to balance global and local features, improving robustness to lesion variability and efficiency in resource-limited environments. (3) A learnable DropPath regularization strategy is applied to enhance generalization under small-sample conditions.

## 2 Materials and methods

### 2.1 Image source and acquisition

The study collected images from the high-quality strawberry planting park in Jinhe Town, Hohhot City, Inner Mongolia Autonomous Region, chosen for its concentrated and representative strawberry cultivation. The research team systematically acquired diverse images of healthy and diseased strawberries in March 2025.

**Figure 1:**
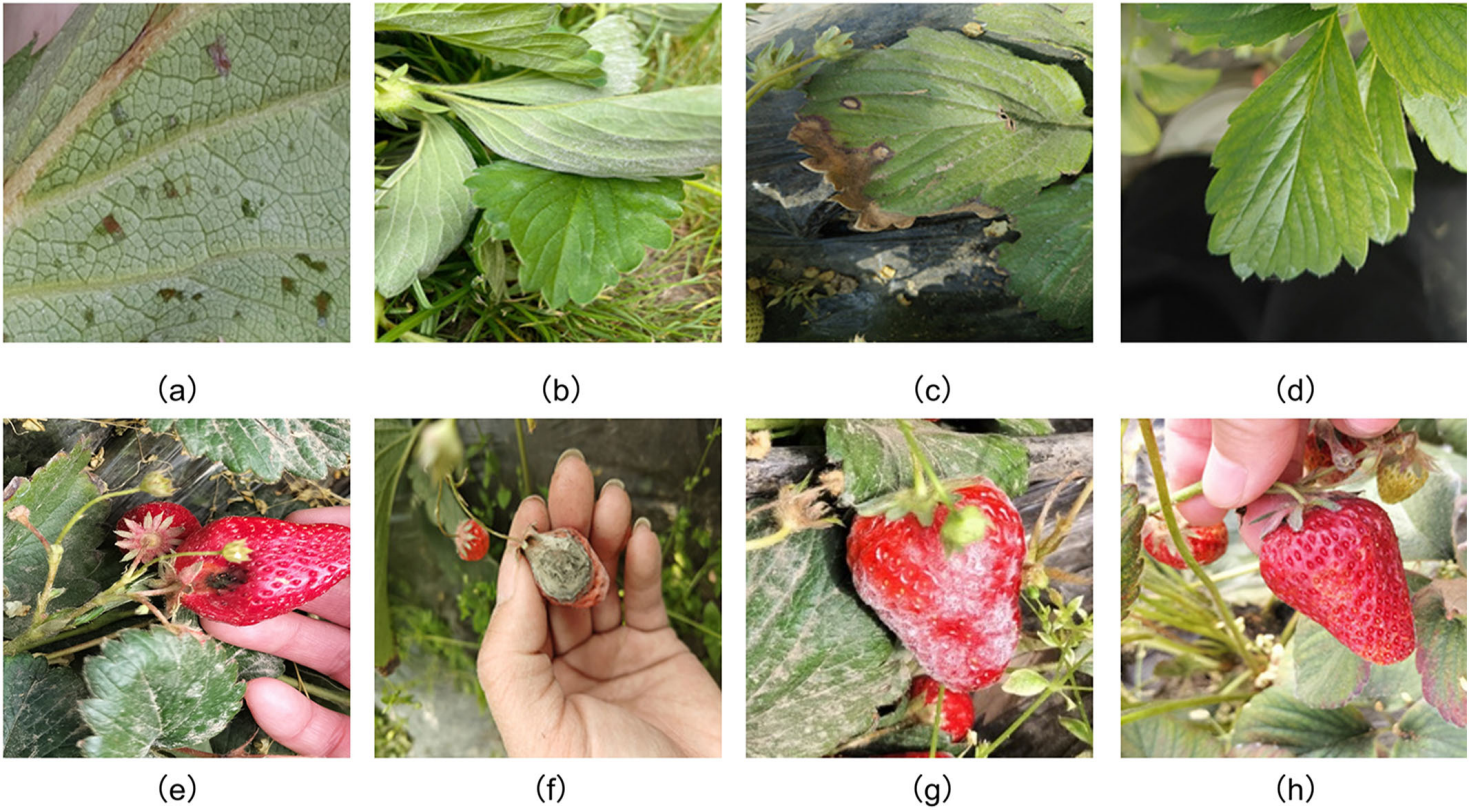
Healthy and diseased strawberry leaves and fruits **(a)** angular_leafspot **(b)** powdery_mildew_leaf **(c)** leaf_spot **(d)** healthy_leaf **(e)** anthracnose_fruit_rot **(f)** gray_mold **(g)** powdery_mildew_fruit **(h)** healthy_ripe_fruit.

During the image acquisition phase, the research team selected the OPPO OnePlus Ace3 smartphone as the capturing device. All images were saved at a resolution of 4096 × 3512 pixels, ensuring sufficient clarity for lesion identification. In total, 1,486 images were obtained, including both healthy and diseased strawberry leaves and fruits. The dataset covers six major disease types: angular leaf spot, powdery mildew, and leaf spot on leaves, and anthracnose, powdery mildew, and gray mold on fruits, along with corresponding healthy samples. Representative examples are shown in **Figure 1**.

### 2.2 Data preprocessing

In this study, a systematic data preprocessing workflow was developed to ensure that the strawberry disease image dataset was high-quality, representative, and suitable for deep learning model training. The workflow included image selection, data augmentation, and class balancing with uniform resizing, providing a robust foundation for subsequent model development.

First, raw images were first carefully screened to remove non-compliant samples, including those with abnormal lighting, motion blur, or defocus, and visual interference such as occlusions or reflections. Following this initial cleaning, images were labeled based on authoritative references, such as the *Strawberry Pest and Disease Diagnosis and Control Atlas*, and with guidance from strawberry experts to ensure accurate category assignment. All labeled results were then cross-checked by two experts, with disputed images either re-evaluated or removed, thereby guaranteeing both the consistency and reliability of the annotations. This process resulted in 1,348 high-quality images that preserved the diversity and representativeness of the dataset.

**Figure 2:**
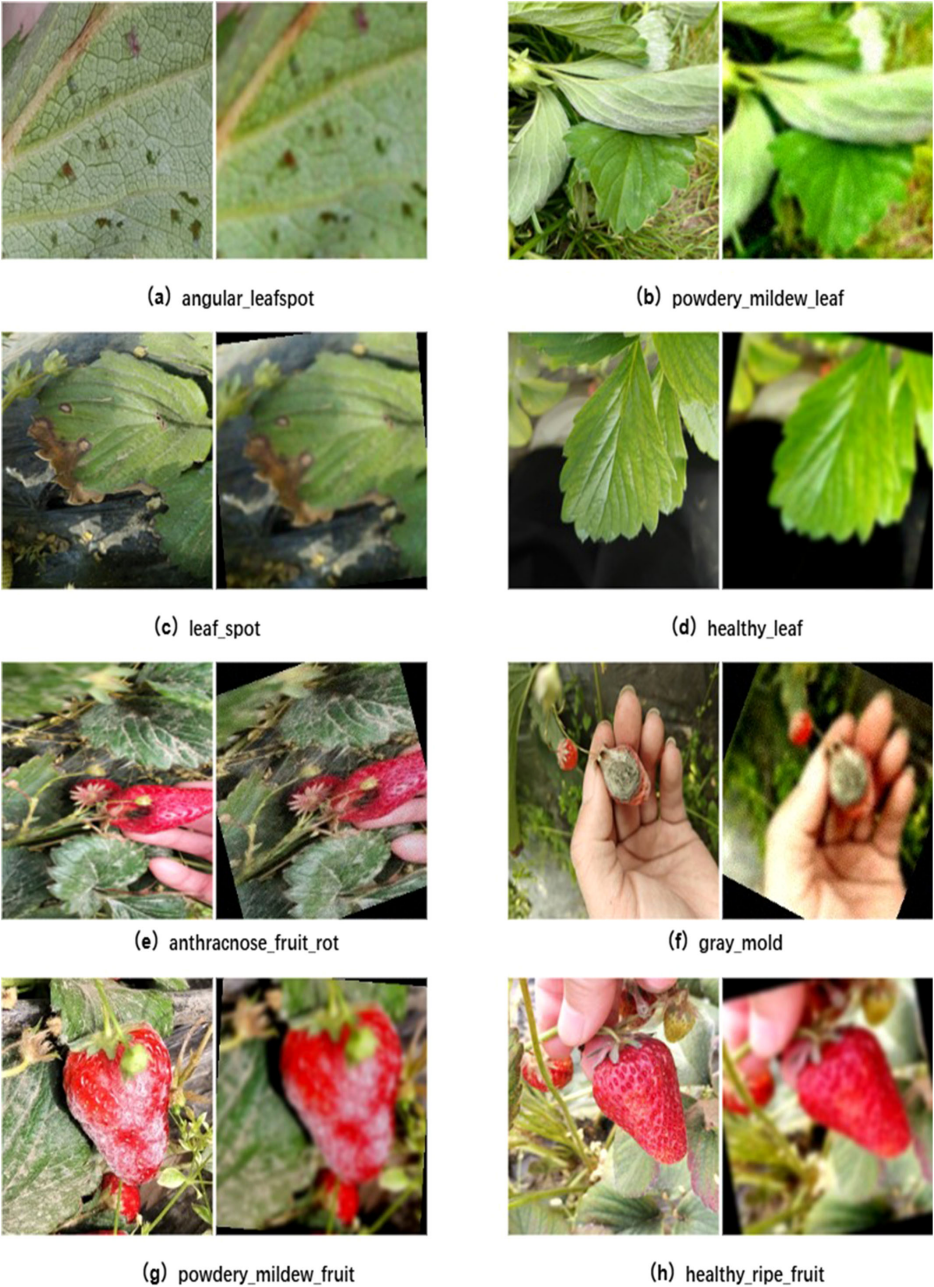
Data augmented healthy and diseased strawberry leaves and fruits **(a)** angular_leafspot **(b)** powdery_mildew_leaf **(c)** leaf_spot **(d)** healthy_leaf **(e)** anthracnose_fruit_rot **(f)** gray_mold **(g)** powdery_mildew_fruit **(h)** healthy_ripe_fruit.

Secondly, to address the limited number of filtered images, data augmentation was applied to expand the training dataset. Data augmentation artificially expands the training dataset through various transformations, and has been shown to effectively mitigate model overfitting (Khosla and Saini, 2020; Maharana et al., 2022). Geometric transformations included random rotations (–45° to +45°), horizontal flips (50% probability), and random cropping from 200×200 regions followed by resizing to 224×224 pixels. Image quality was enhanced with Gaussian blur (kernel radius 0.5–1.5) and Gaussian noise (σ = 10), while color adjustments varied brightness (0.7–1.3×), contrast (0.8–1.2×), and saturation (0.6–1.4×). In each augmentation cycle, two to three transformations were randomly combined to generate two distinct augmented versions per original image, increasing dataset diversity and improving model generalization. Furthermore, class imbalance was addressed using Scikit-learn’s resample method. Images were organized by category and sampled with or without replacement to achieve target class sizes. Oversampling ensured adequate representation for underrepresented categories, whereas downsampling prevented bias from overrepresented classes. All images were subsequently resized to 224×224 pixels and converted to RGB format, ensuring consistent input for model training. **Figure 2** illustrates original and augmented images for healthy and diseased strawberries, with the original images on the left and the augmented images on the right.

**Figure 3:**
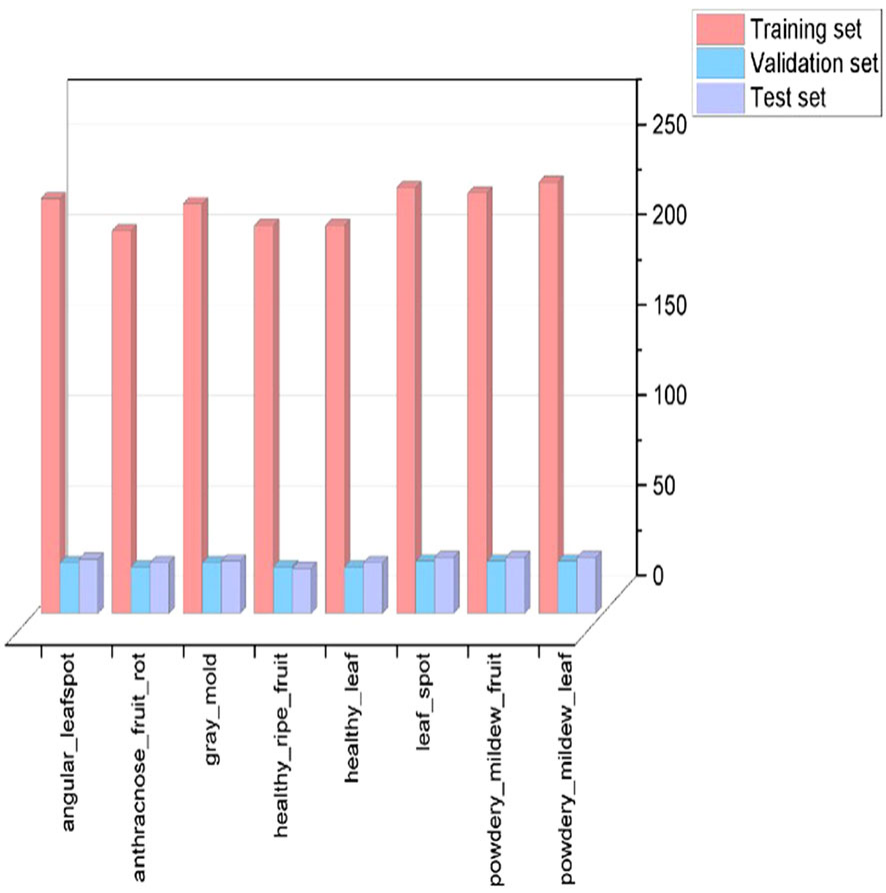
The number of training, validation, and test sets for different diseases.

The data augmentation process expanded the dataset to 2,261 samples (**Table 1**). Stratified random sampling divided the dataset into training, validation, and test sets in an 8:1:1 ratio. The training set facilitated feature learning and parameter optimization, the validation set guided hyperparameter tuning and mitigated overfitting, and the test set provided an independent evaluation of model performance and practical applicability. Dataset partitioning is shown in **Figure 3**.

**Table 1:** Strawberry disease dataset.

Category name	Category label	Original dataset	Augmented dataset
angular_leafspot	0	146	288
powdery_mildew_leaf	1	157	299
leaf_spot	2	158	296
healthy_leaf	3	142	269
anthracnose_fruit_rot	4	143	266
powdery_mildew_fruit	5	286	293
gray_mold	6	191	284
healthy_ripe_fruit	7	125	266
sum	8	1348	2261

## 3 Strawberry disease classification algorithm model

### 3.1 EfficientNet network architecture

**Figure 4:**
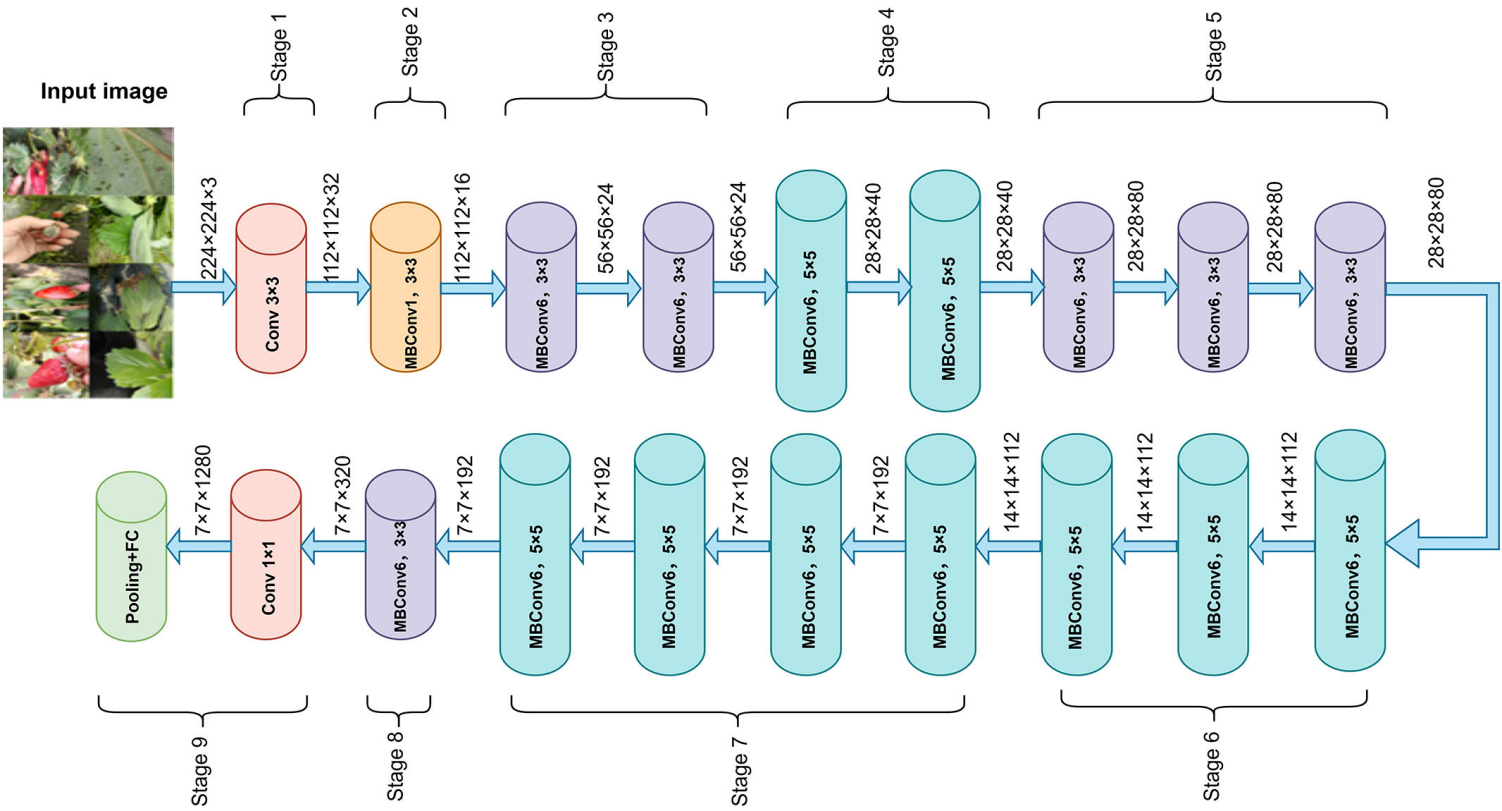
EfficientNet network architecture diagram.

**Figure 5:**
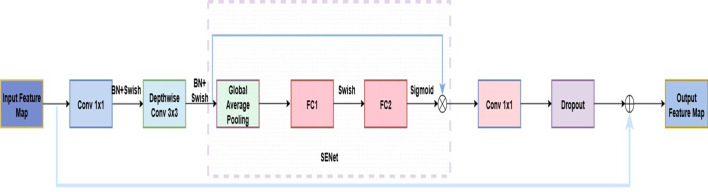
MBConv architecture diagram.

In recent years, CNNs have achieved remarkable success in plant disease recognition and dominate agricultural image analysis (Abade et al., 2021). Classic architectures such as AlexNet, VGGNet, and ResNet (He et al., 2016) are widely used due to their strong feature extraction capabilities. The EfficientNet series is a lightweight architecture that jointly scales network width, depth, and input resolution, reducing complexity while maintaining high accuracy (Tan and Le, 2019). This makes it well-suited for resource-constrained field applications (Wei et al., 2022; Wang et al., 2023). Among them, EfficientNetB0, balancing accuracy and efficiency, has shown strong performance in plant disease recognition (Liu et al., 2020b) and was selected as the backbone for the strawberry disease model. EfficientNetB0 consists of nine stages. Stage 1, also called “stem_conv,” serves as the network’s input stage. It applies a 3×3 convolutional layer, followed by batch normalization (BN) (loffe and Szegedy, 2015) and the Swish activation (Ramachandran et al., 2017), performing the initial spatial downsampling and feature mapping. Stages 2–8 stack MBConv blocks (Sandler et al., 2018) for feature extraction, while Stage 9 forms the classification head with a 1×1 convolution, BN, Swish, average pooling, and a fully connected layer. Each MBConv block includes a 1×1 expansion convolution, depthwise separable convolution (Chollet, 2017), squeeze-and-excitation module, 1×1 reduction convolution, and Dropout to reduce overfitting. The overall architecture is shown in **Figure 4**, with the MBConv block detailed in **Figure 5**.

### 3.2 ENet-CAEM network architecture

**Figure 6:**
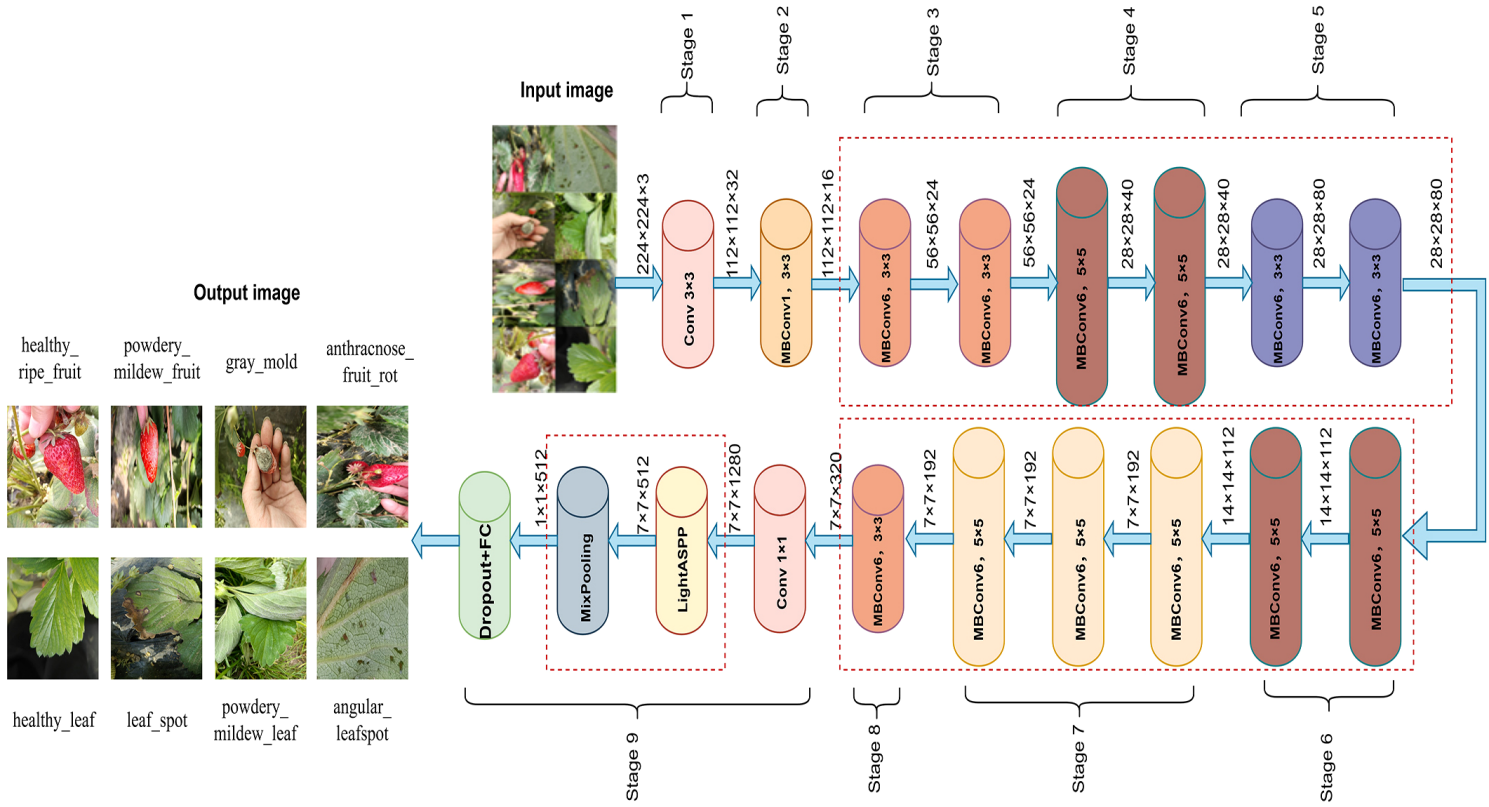
ENet-CAEM network architecture diagram.

**Figure 7:**
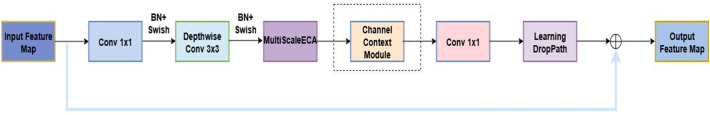
Improved MBConv Architecture Diagram.

Based on EfficientNetB0, this study proposes an enhanced network architecture, ENet-CAEM, to address key challenges in strawberry disease recognition, including complex background interference, lesion scale diversity, and limited generalization under small sample conditions. The overall architecture is shown in **Figure 6**. Through several multi-level improvements, the model significantly enhances disease recognition performance in complex agricultural environments. It consists of the initial stem_conv layer followed by a sequence of MBConv blocks. The enhancements are as follows:

1. Within each MBConv block, the original SE module is replaced with the MultiScaleECABlock, which captures lesion features at different scales via parallel multi-branch convolutions (3×3, 5×5, 7×7), it also incorporates a dynamic weight fusion mechanism to adjust feature importance across scales.
2. In Stages 4–6, a Channel Context Module (CCM) is embedded after the depthwise convolution of each MBConv block to model channel-level contextual information and enhance focus on critical lesion features. To improve mobile deployment efficiency, the number of MBConv blocks is reduced from 16 to 13, lowering computational complexity while maintaining performance.
3. A Learnable DropPath mechanism is applied to MBConv residual connections, a dynamic weight fusion mechanism that adaptively adjusts the importance of features across scales.
4. After MBConv feature extraction, a lightweight Atrous Spatial Pyramid Pooling (LightASPP) module is integrated. Its output passes through a Mixed Pooling layer, combining average and max pooling, followed by a Dropout layer and a fully connected layer to map features to disease categories. The improved MBConv structure is shown in **Figure 7**.

#### 3.2.1 MultiScale efficient channel attention

**Figure 8:**
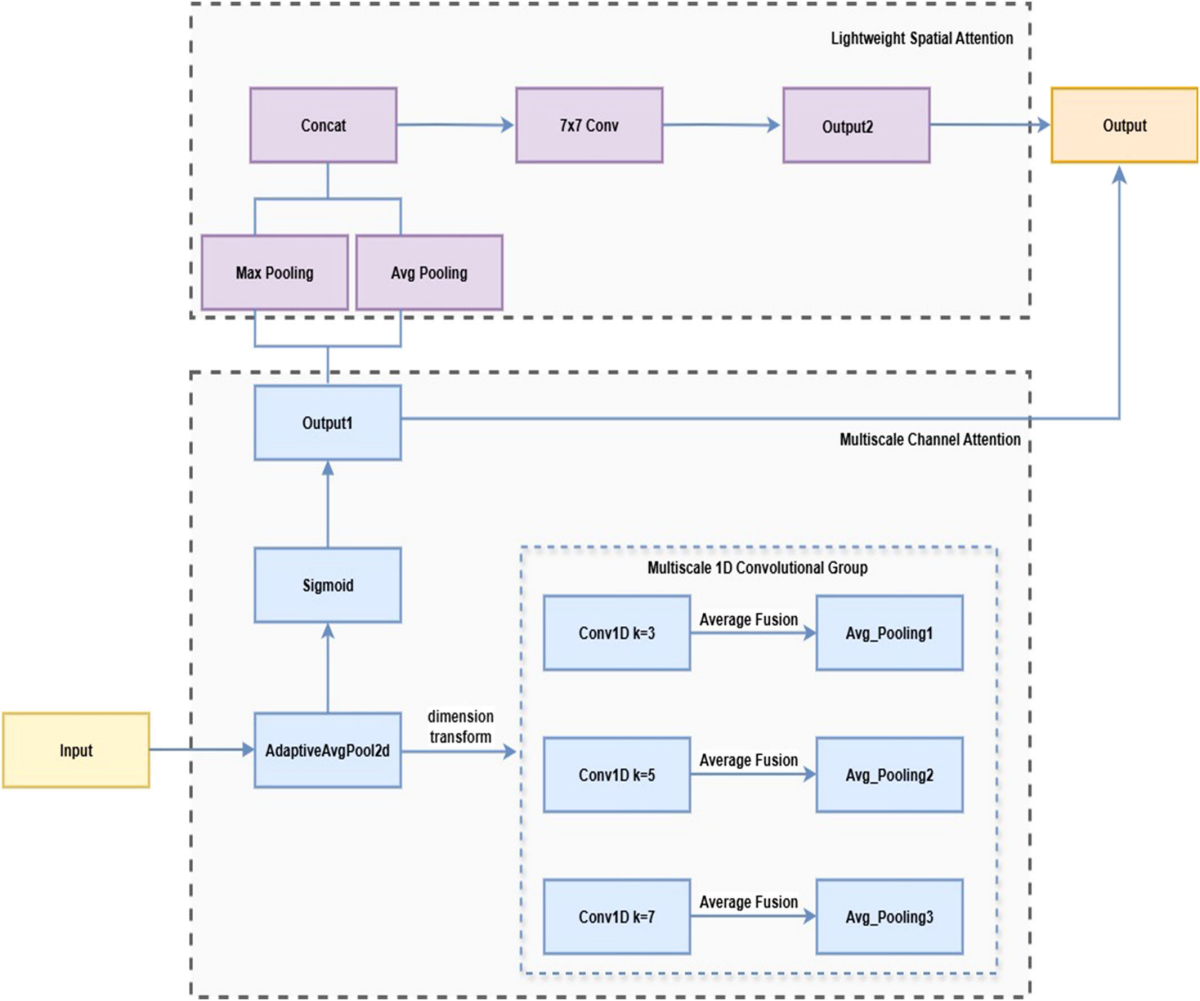
Multi-scale efficient channel attention.

To improve the model’s ability to detect lesions of varying sizes, this paper proposes the MultiScaleECA module based on the Efficient Channel Attention (ECA) mechanism (Wang et al., 2020). While ECA efficiently captures inter-channel relationships using 1D convolutions, single-scale kernels limit adaptability to large lesion size variations. MultiScaleECA employs multiscale 1D convolutions to model channel dependencies across different receptive fields. It also integrates a lightweight spatial attention mechanism to enhance focus on edges and textures, improving lesion localization and suppressing background noise. The overall architecture is shown in **Figure 8**, with pseudocode in **Algorithm 1**. The module consists of two main components:

Algorithm 1

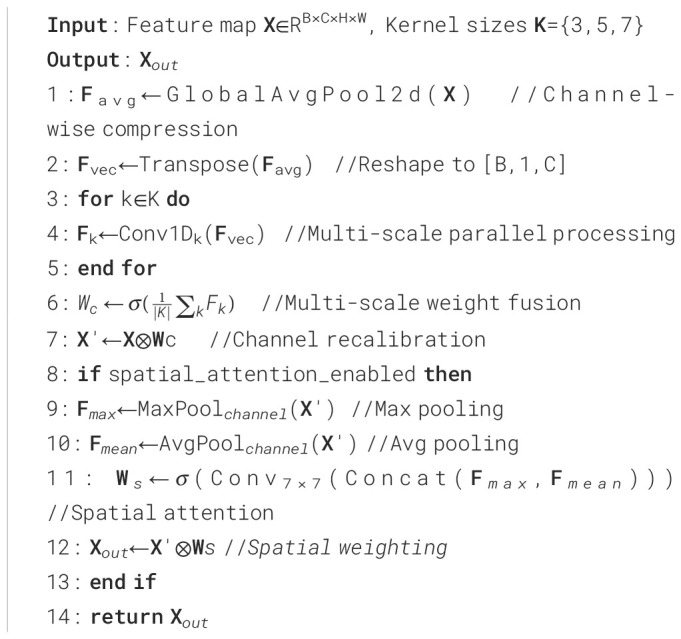

1. Multiscale Channel Attention

To enhance sensitivity to lesions of varying sizes, the module models channel dependencies across multiple scales through multiscale one-dimensional convolutions. The computation is as follows (see Equations 1–6):

1. Global average pooling is applied to the input feature map $X\in R^{B\times C\times H\times W}$ (Lin et al., 2013) to extract channel-wise statistics while compressing spatial dimensions:

(1) $$\begin{array}{c} F_{avg}=\text{AvgPool}(X)\in R^{B\times C\times 1\times 1} \end{array}$$

Here, B is the batch size, C  is the number of channels, and H,W are spatial dimensions. This operation captures global semantic information, enhancing the model’s ability to recognize lesions of varying sizes.

2. To adapt to subsequent one-dimensional convolution modeling, $\;F_{vec}$ is transposed to form a channel sequence:

(2) $$\begin{array}{c} F_{vec}=\text{Transpose}(F_{avg})\in R^{B\times 1\times C} \end{array}$$

This operation reshapes the feature representation, enabling effective modeling of inter-channel dependencies through subsequent 1D convolutions.

3. To capture multiscale channel context, parallel 1D convolutions with kernel sizes of 3, 5, and 7 are applied to $F_{vec}$:

(3) $$\begin{array}{c} F(k)=Conv1Dk(F_{vec}),k=3,5,7 \end{array}$$

F(k) represents the channel attention at each scale, enabling the model to capture dependencies across channels and better detect lesions of varying sizes.

4. To integrate the multiscale channel information, the above multi-scale convolution results are fused through averaging:

(4) $$\begin{array}{c} F_{ms}=\frac{1}{k}\sum_{i=1}^{n}Conv1Dk(F_{vec}),n=3 \end{array}$$

Here, n=3 denotes three scales, and $F_{ms}$ is the fused multi-scale channel response, enhancing attention robustness by aggregating information across scales.

5. To obtain the importance score for each channel, use Sigmoid activation to generate a weight vector for the fusion result:

(5) $$\begin{array}{c} W_{c}=\sigma (F_{ms})\in R^{B\times C\times 1\times 1} \end{array}$$

σ(·) denotes the sigmoid function, and $W_{c}$ represents the attention weights of each channel, indicating its importance for the current image and guiding channel-wise feature modulation.

6. Finally, the generated channel weights are multiplied by the original input feature map in a channel-wise manner to perform channel recalibration:

(6) $$\begin{array}{c} X^{\prime }=X\cdot W_{c} \end{array}$$

*X*′ is the weighted feature map, enhancing informative features, suppressing redundant channels, and improving the model’s ability to distinguish lesions of different sizes.

2. Lightweight Spatial Attention

To capture spatial characteristics such as lesion edges and textures, a lightweight spatial attention module is added after channel attention. Max pooling and average pooling along the channel dimension produce a two-channel feature map, which is concatenated. This computation is detailed in Equation 7:

(7) $$\begin{array}{c} F_{\text{spatial}}=\text{Concat}[{\text{AvgPool}}_{c}(X^{\prime }),{\text{MaxPool}}_{c}(X^{\prime })]\in R^{B\times 2\times H\times W} \end{array}$$

${\text{AvgPool}}_{c}(X^{\prime })\;$ and ${\text{MaxPool}}_{c}(X^{\prime })$ denote average and max pooling along the channel dimension, and $F_{\text{spatial}}\;$ is their two-channel concatenation, capturing spatial features for blurred or overlapping lesions.

(8) $$\begin{array}{c} W_{s}=\sigma ({\text{Conv}}_{7\times 7}(\text{Fspatial}))\in R^{B\times 2\times H\times W} \end{array}$$

A 7×7 convolution followed by Sigmoid activation generates the spatial weight map $W_{s}$, as defined in Equation 8, which is applied to $X^{\prime }$ to enhance lesion regions, suppress background, and improve recognition accuracy and robustness (Woo et al., 2018).

#### 3.2.2 Channel context module

**Figure 9:**
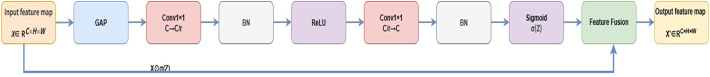
CCM module structure diagram.

To improve robustness against complex background interference, a Channel Context Module (CCM) is added in intermediate stages. Inspired by the SE mechanism (Hu et al., 2018), it uses global channel statistics to recalibrate features, thereby enhancing lesion regions and suppressing background noise. For input $X\in {\mathbb{R}}^{C\times H\times W}$, global average pooling produces a context vector, which passes through a 1×1 convolution to reduce channels to C/r with ReLU, then a second 1×1 convolution restores channels to C. Sigmoid activation generates context-aware weights σ(Z), applied to X via element-wise multiplication to yield $X^{\prime }$. Batch normalization is applied during compression and reconstruction for stability. **Figure 9** shows the structure, and **Algorithm 2** provides pseudocode.

Algorithm 2 **Channel context module.**

**Input**: Feature map **X**∈R^C×H×W^, Reduction ratio *r***Output**: **X**'
1: **F**_gap_←GlobalAvgPool2d(**X**)  //Channel-wise statistics
2: **Z**_1_**←**Conv1x1(C, C/r)(**F**_gap_)  //Compress to C/r channels
3: **Z**_1_←BatchNorm(**Z**_1_)   //Stabilize training
4: **Z**_1_**←**ReLU(**Z**_1_)     //Add nonlinearity
5: **Z**_2_←Conv1x1(C/r,C)(**Z**_1_)  //Recover original channels
6: **Z**_2_←BatchNorm(**Z**_2_)  //BN for reconstruction
7: **W**_c_**← σ(Z**_2_)     //Sigmoid attention weights
8: **X**'← **X** ⊗ **W**_c_    //Channel-wise scaling
9: **return X**'

#### 3.2.3 Lightweight atrous spatial pyramid pooling

In strawberry disease detection, lesions often exhibit multi-scale variation, diffusion, and morphological diversity, challenging traditional convolutions in capturing spatial context. The LightASPP module uses a streamlined set of multi-scale dilated convolutions to expand the receptive field and enhance multi-scale representation while controlling computational cost. Compared with standard ASPP (Chen et al., 2017), LightASPP introduces three lightweight branches with dilation rates of 3, 6, and 9, each comprising a 3×3 dilated convolution followed by BN and ReLU, omitting redundant 1×1 convolutions. The global average pooling path is retained, with output channels compressed to 128 and aligned via bilinear interpolation, preserving global context efficiently. Multi-scale features are fused through direct concatenation without an additional 1×1 convolution.

**Figure 10:**
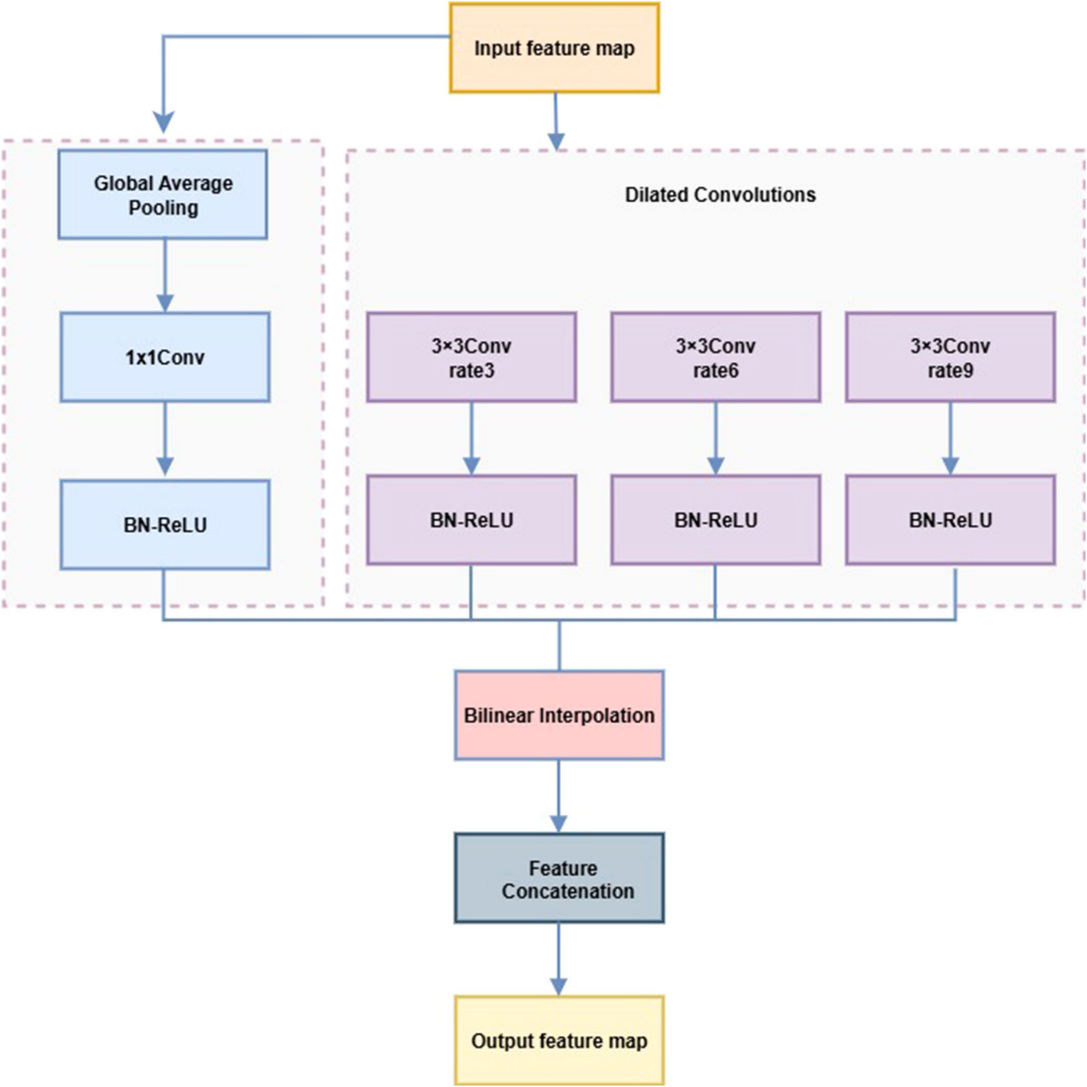
LightASPP architecture diagram.

This module is integrated into the top feature layer of EfficientNetB0 to enhance adaptability to complex lesion morphology while maintaining a lightweight design. The small (rate=3), medium (rate=6), and large (rate=9) dilation branches capture early, typical, and diffuse lesions, respectively, while the global pooling path improves recognition of densely distributed spots. The LightASPP structure is shown in **Figure 10**, and its pseudocode is provided in **Algorithm 3**.

Algorithm 3 **LightASPP module.**

**Input**: Feature map **X**∈R^B×C×H×W^, Atrous rates R={3,6,9}
**Output**: **X'**
1: **F**_list_**←**empty list    //Initialize feature storage
2: **for** r∈R **do**     // Process each dilated branch
3:  **F**_r_**←**Conv3x3_dilation=r(**X**) // 3×3 dilated conv (pad   = r)
4:  **F**_r_**←**BN**(F**_r_)
5:  **F**_r_**←**ReLU**(F**_r_)
6:  **F**_list.append(_**_F_**_r)_     // Add to feature list
7: **end for**
8: **F**_gap_**←**GlobalAvgPool(**X**)  // Global context [1×1]
9: **F**_gap_**←**1×1 Conv(**F**_gap_) to reduce channels to 128  //Compress to 128 channels
10: **F**_gap_**←**BN**(F**_gap_)
11: **F**_gap_**←**ReLU**(F**_gap_)
12: **F**_gap_**←**BilinearUpsample(**F**_gap_) to size H×W  // Bilinear interpolation
13: **F**_list.append(_**_F_**_gap)_    // Add global feature
14: **X'←**Concat**(F**_list_)   // Channel-wise concatenation
15: **return X'**

#### 3.2.4 Mixed pooling

To enhance feature extraction flexibility and robustness, a Mixed Pooling strategy is introduced. Traditional pooling methods—max and average pooling—each have limitations: max pooling highlights salient features but neglects global context, while average pooling preserves overall structure but weakens local detail sensitivity. Mixed Pooling resolves this trade-off through a learnable weighted fusion of both operations, controlled by a trainable parameter λ∈[0,1], which adaptively balances local and global information. It also complements regularization methods such as data augmentation, dropout, and weight decay, improving model generalization (Gu et al., 2018). The formulation is given in Equation 9 (Yu et al., 2014):

(9) $$\begin{array}{c} y_{kij}=\lambda \cdot \underset{(p,q)\in R_{ij}}{\max}x_{kpq}+(1-\lambda )\cdot \frac{1}{\left|R_{ij}\right|}\sum_{(p,q)\in R_{ij}}x_{kpq} \end{array}$$

Here, λ determines the contribution of max and average pooling, enabling the network to adaptively learn the optimal pooling strategy for varying feature scales and data distributions. This dynamic mechanism enhances the model’s robustness and adaptability across complex visual tasks.

#### 3.2.5 Learnable DropPath

DropPath is a regularization strategy that randomly removes network paths—such as residual connections. This process effectively creates an implicit ensemble and enhances model generalization (Huang et al., 2016). In the traditional DropPath method, paths are dropped with a fixed probability set by hyperparameters, typically applied uniformly across all layers.

To achieve finer control over path activation during training, the Learnable DropPath variant assigned to each block an individually learnable drop probability (Tan and Le, 2021). This adaptive mechanism allows the model to dynamically adjust path importance. It overcomes the rigidity of fixed drop rates, thereby enhancing both training flexibility and overall model performance.

## 4 Results and analysis

### 4.1 Experimental environment and parameter settings

Experiments were conducted on a Windows 11 system with an Intel Xeon Gold 6330 CPU and an NVIDIA RTX 3090 GPU, implemented in PyTorch 2.4.1 with CUDA 12.8. The model was trained for 200 epochs using the Adam optimizer with a batch size of 32. To avoid convergence issues caused by a fixed learning rate, a cosine annealing schedule was adopted for dynamic learning rate adjustment.

### 4.2 Evaluation metrics

In this study, model performance is analyzed through four commonly used metrics: Accuracy, Precision, Recall, and F1 Score. Ahmed and Yadav pointed out that these metrics are of significant importance for evaluating plant disease recognition models (Ahmed and Yadav, 2024). The definitions and calculation formulas are presented in Equations 10–13 (Fawcett, 2006):

1. Accuracy: the percentage of samples that the model predicts correctly from the total set of samples:

(10) $$\begin{array}{c} \text{Accuracy}=\frac{TP+TN}{TP+TN+FP+FN} \end{array}$$

2. Precision: the proportion of predicted positive samples that are actually positive:

(11) $$\begin{array}{c} \text{Precision}=\frac{TP}{TP+FP} \end{array}$$

3. Recall: the proportion of samples that are actually positive and are correctly predicted as positive samples:

(12) $$\begin{array}{c} Recall=\frac{TP}{TP+FN} \end{array}$$

4. F1 Score: the balance between precision and recall, calculated as their harmonic mean.

(13) $$\begin{array}{c} F1\;Score=\frac{2\times Precision\times Recall}{Precision+Recall} \end{array}$$

Here, TP denotes the number of positive samples correctly identified as positive, TN denotes the number of negative samples correctly identified as negative, FP denotes the number of negative samples incorrectly identified as positive, and FN denotes the number of positive samples incorrectly identified as negative.

### 4.3 Comparative experiment

#### 4.3.1 Model performance comparison on self-built dataset

To evaluate the performance of the proposed model, we compared ENet-CAEM with several classic architectures, including AlexNet, VGG16, ResNet, GoogleNet, MobileNetV2, MobileNetV3-Small, RegNet (Xu et al., 2022), ConvNeXt (Liu et al., 2022), and MobileViT (Mehta and Rastegari, 2021). The results are presented in **Table 2**. (Dakwala et al., 2022) pointed out that CNN architectures vary significantly in fruit classification performance, providing the basis for our comparison. As shown in **Table 2**, ENet-CAEM outperformed traditional CNNs on the strawberry dataset, improving accuracy and recall by 4.29% and 4.09% over EfficientNetB0, with only a 2.53 MB increase in parameters.

**Table 2:** Performance comparison of self-built dataset on different classification networks.

Model	Accuracy (%)	Precision (%)	Recall (%)	F1Score (%)	Params (MB)	Flops (GB)
AlexNet	69.00	69.00	69.00	69.00	14.60	0.310
ResNet	76.00	78.00	76.00	75.00	21.29	3.680
VGG16	76.00	77.00	76.00	76.00	134.29	15.470
GoogleNet	81.12	83.45	80.70	80.72	5.61	3.021
MobileNetV2	79.00	81.00	79.00	78.00	2.23	0.319
MobileNetV3-small	81.00	83.00	81.00	81.00	1.53	0.060
RegNet	81.55	82.52	81.26	81.35	2.32	0.249
ConvNeXt	82.83	83.61	82.58	82.70	27.80	4.450
MobileViT	83.26	84.75	82.99	83.39	0.95	0.270
EfficientNetB0	81.55	81.60	81.50	81.52	4.02	0.410
ENet-CAEM	85.84	86.53	85.59	85.75	6.55	0.461

As shown in **Table 3**, ENet-CAEM consistently outperformed other models in strawberry disease recognition. For leaf diseases, it achieved accuracies of 89.66%, 86.21%, and 83.87% for angular leaf spot, powdery mildew, and leaf spot, respectively. It ranked among the top across all models, indicating strong discriminative capability in handling leaf diseases characterized by complex lesion morphology and blurred boundaries. For fruit diseases, accuracies reached 88.89% for anthracnose, 89.66% for gray mold, and 75.61% for fruit powdery mildew. This indicated that the improved model possessed high feature sensitivity and generalization capability. It effectively handled complex characteristics such as small spots, blurred diffusion, and powder-like textures on diseased fruit surfaces. Healthy samples were also accurately classified, with 92.00% and 86.36% accuracy for healthy leaves and fruits, respectively. This performance remained consistently high across all models and effectively reduced the risk of misclassifying healthy samples as diseased.

**Table 3:** Strawberry disease identification results.

Model	Test set precision							
	Angular_ leafspot (%)	Powdery_ mildew_ leaf (%)	Leaf_spot (%)	Healthy_ leaf (%)	Anthracnose_ fruit_ rot (%)	Gray_mold (%)	Powdery_ mildew_ fruit (%)	Healthy_ ripe_ fruit (%)
AlexNet	58.00	73.00	78.00	70.00	73.00	63.00	65.00	72.00
ResNet	79.00	79.00	78.00	95.00	77.00	84.00	67.00	61.00
VGG16	74.00	87.00	68.00	86.00	77.00	79.00	66.00	76.00
GoogleNet	77.78	86.67	100.00	92.59	75.00	82.14	65.91	87.50
MobileNetV2	76.00	72.00	82.00	92.00	81.00	79.00	70.00	92.00
MobileNetV3-small	84.00	96.00	77.00	86.00	69.00	86.00	71.00	93.00
RegNet	83.33	83.33	80.00	88.00	88.00	82.76	69.05	85.71
ConvNeXt	78.79	92.86	81.48	88.00	85.19	92.86	71.43	78.26
MobileViT	89.29	83.33	92.00	96.00	89.29	92.31	61.90	73.91
EfficientNetB0	89.66	93.10	77.42	89.66	70.00	82.76	74.19	76.00
ENet-CAEM	89.66	86.21	83.87	92.00	88.89	89.66	75.61	86.36

Overall, ENet-CAEM achieved superior precision and generalization compared to AlexNet, EfficientNet, MobileNetV3, and RegNet, demonstrating enhanced robustness and reliability under complex agricultural conditions.

#### 4.3.2 Comparison with existing methods on self-built datasets

To comprehensively evaluate the effectiveness and advanced capabilities of ENet-CAEM in strawberry disease identification, we compared it with three recent state-of-the-art models: G-ResNet50, T-CNN, and MS-DNet. These models were fairly compared against our proposed ENet-CAEM model using the same strawberry disease dataset. **Table 4** presented the performance metrics of each model on the same test set.

**Table 4:** Comparison with existing methods on self-built datasets.

Model	Accuracy (%)	Precision (%)	Recall (%)	F1Score (%)	Params (MB)	Flops (GB)
G-ResNet50	77.68	80.28	77.68	77.22	25.57	4.134
T-CNN	84.98	86.83	85.17	85.02	25.87	4.494
MS-DNet	80.69	81.97	80.52	80.72	6.01	0.973
ENet-CAEM	85.84	86.53	85.59	85.75	6.55	0.461

As shown in **Table 4**, G-ResNet50 (Wenchao and Zhi, 2022) introduced Focal Loss and PlantVillage pre-trained weights, but showed relatively low performance on our field dataset, with high parameter count and computational cost, indicating limited generalization and efficiency in complex field scenarios. T-CNN (Wang et al., 2021) proposes a trilinear convolutional architecture that decouples crop identification from disease detection, aiming to capture finer features through bilinear pooling. It achieved high accuracy and F1 scores on our dataset, ranking second only to ENet-CAEM. However, its high model complexity severely limits its deployment potential on resource-constrained mobile or embedded devices.MS-DNet (Chen et al., 2022) uses depthwise separable convolutions and SE modules to reduce complexity while maintaining moderate performance, but its accuracy and F1 score lag behind ENet-CAEM, reflecting trade-offs in feature extraction.

In contrast, ENet-CAEM achieved superior performance while maintaining efficiency. Its parameter count was comparable to lightweight MS-DNet and far lower than G-ResNet50 and T-CNN, and its computational complexity was the lowest, with a 52.6% reduction compared to MS-DNet, demonstrating the effectiveness of its architectural improvements.

#### 4.3.3 Generalization ability verification on public datasets

To comprehensively evaluate the generalization performance of the ENet-CAEM model, this study conducted rigorous cross-dataset testing on the publicly available PlantVillage strawberry disease dataset from Kaggle. The dataset contains 2,500 high-quality images across seven strawberry disease categories: powdery_mildew_leaf, anthracnose_fruit_rot, leaf spot, blossom blight, angular_leafspot, gray mold, and powdery_mildew_fruit. The dataset differs from the self-built one in data distribution and acquisition conditions, enabling assessment of the model’s robustness under unseen scenarios. The results are shown in **Table 5**.

**Table 5:** Performance comparison of public datasets on different classification networks.

Model	Accuracy (%)	Precision (%)	Recall (%)	F1Score (%)	Params (MB)	Flops (GB)
AlexNet	73.00	71.00	67.00	66.00	14.60	0.310
ResNet	82.00	83.00	79.00	79.00	21.29	3.680
VGG16	89.00	86.00	83.00	84.00	134.29	15.470
GoogleNet	86.66	91.59	82.49	85.11	5.61	3.021
MobileNetV2	88.00	88.00	79.00	81.00	2.23	0.319
MobileNetV3-small	88.00	85.00	84.00	84.00	1.53	0.060
RegNet	89.00	87.00	81.00	83.00	2.32	0.249
ConvNeXt	94.74	93.99	91.69	92.59	27.80	4.450
MobileViT	92.18	90.88	88.73	89.53	0.95	0.270
EfficientNetB0	93.94	92.29	89.76	90.78	4.02	0.410
ENet-CAEM	97.39	94.95	92.49	93.60	6.55	0.461

ENet-CAEM achieved the highest accuracy, precision, recall, and F1 score among all compared models, and maintained a lower parameter count and computational complexity. These findings confirm that the proposed improvements effectively enhance model performance and efficiency.

### 4.4 Ablation experiment

We conducted ablation experiments to assess the contribution of each module in ENet-CAEM, including MultiScaleECA, CCM, Learnable DropPath, LightASPP, and Mixed Pooling. All experiments used identical settings, with EfficientNetB0 as the baseline. The results are presented in **Table 6**.

**Table 6:** Ablation experiment comparison of ENet-CAEM model.

Model	Accuracy (%)	Precision (%)	Recall (%)	F1Score (%)	Params (MB)	Flops (GB)
EfficientNetB0	81.55	81.60	81.50	81.52	4.02	0.410
EfficientNetB0+MultiScaleECA	81.97	85.01	81.84	82.36	1.97	0.244
EfficientNetB0+MultiScaleECA +CCM	82.40	84.56	82.16	82.65	1.97	0.244
EfficientNetB0+MultiScaleECA +CCM+learnable Droppath	83.26	84.51	82.96	83.03	1.97	0.244
EfficientNetB0+MultiScaleECA +CCM+learnable Droppath+ASPP	83.69	84.40	83.22	83.24	6.55	0.461
ENet-CAEM	85.84	86.53	85.59	85.75	6.55	0.461

As shown in **Table 6**, each module progressively improved model performance. Adding MultiScaleECA increased accuracy from 81.55% to 81.97% and improved the F1 score by 0.84%, demonstrating better multi-scale feature perception and discrimination between visually similar disease features. After integrating the CCM module, accuracy increased to 82.40%, showing that channel-level context modeling improves the network’s ability to capture key lesion features. Adding learnable DropPath raised accuracy to 83.26%, demonstrating that adaptive path dropping mitigates overfitting and adapts to complex lesion patterns. Incorporating LightASPP further increased accuracy to 83.69%, reflecting an enhanced receptive field and better detection of lesion edges and diffusion. Finally, Mixed Pooling achieved the best overall results, with 85.84% accuracy, precision 86.53%, and recall 85.59%—an improvement of 4.29 points in accuracy over the baseline—while parameter count increased modestly from 4.02 MB to 6.55 MB and computation rose by 12.4%. Although ENet-CAEM slightly increases model complexity, it delivers substantial gains in accuracy and robustness, confirming the effectiveness of the proposed modules for strawberry disease recognition under complex conditions.

### 4.5 Cross-validation analysis

We evaluated the robustness and stability of the ENet-CAEM model under limited data conditions through 5-fold cross-validation, as shown in **Table 7**. The model achieved an average accuracy of 85.55%, closely matching the 85.84% accuracy on the independent test set, with only a 0.29% difference. All key metrics showed minimal variation, with standard deviations within ±0.5%, indicating stable performance across folds and confirming the model’s strong feature extraction capability and robustness.

**Table 7:** 5-fold cross-validation results.

Fold	Accuracy (%)	Precision (%)	Recall (%)	F1 Score (%)
1	84.81	85.30	84.81	84.71
2	85.50	85.99	85.50	85.60
3	85.56	85.85	85.56	85.49
4	86.30	86.45	86.30	86.20
5	85.56	86.10	85.56	85.54
Mean±Std	85.55± 0.48	85.94±0.42	85.55±0.48	85.51±0.53

### 4.6 Classification performance evaluation

**Figure 11:**
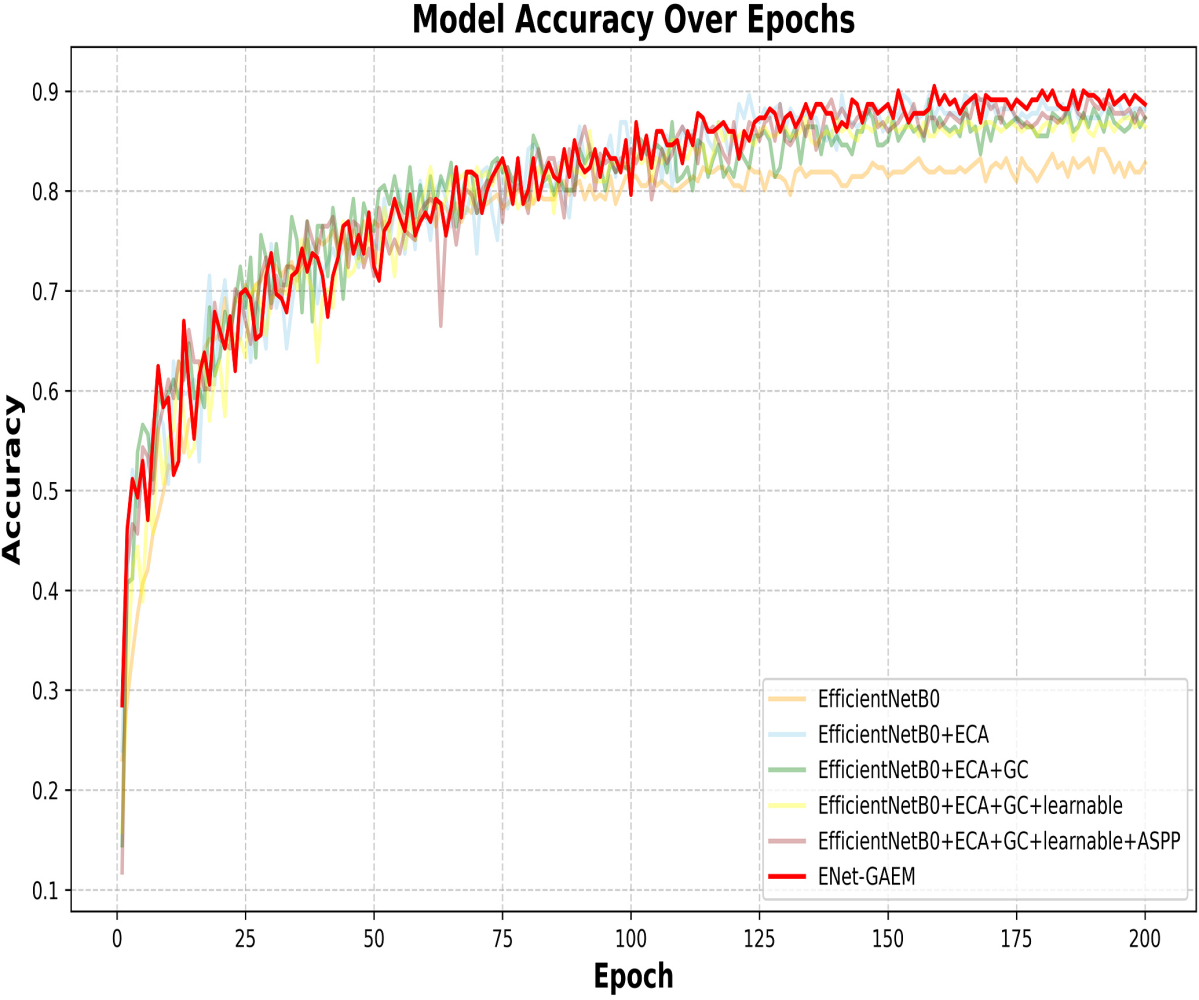
Comparison of validation accuracy across epochs.

**Figure 12:**
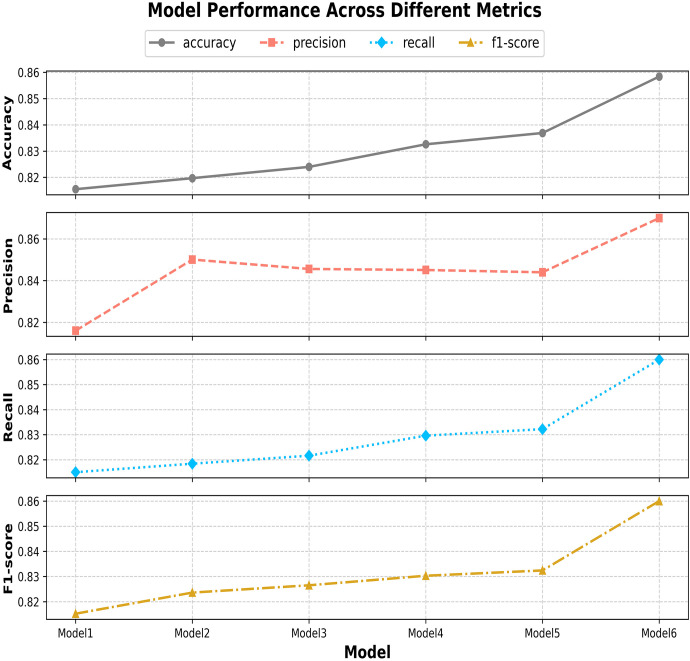
Curves showing changes in various indicators after use of each module.

**Figure 13:**
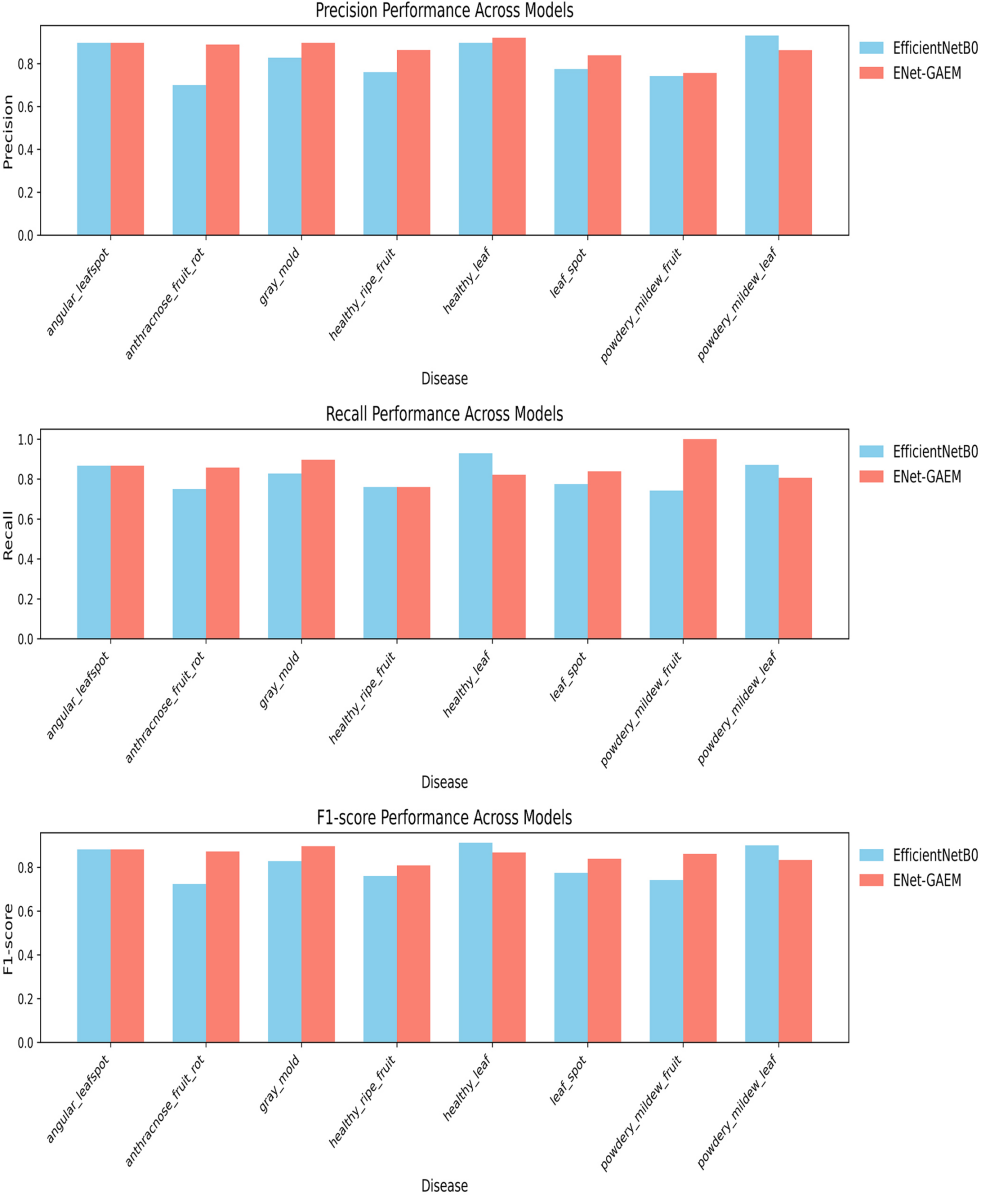
Curves showing changes in various indicators for different diseases before and after model improvement.

**Figure 14:**
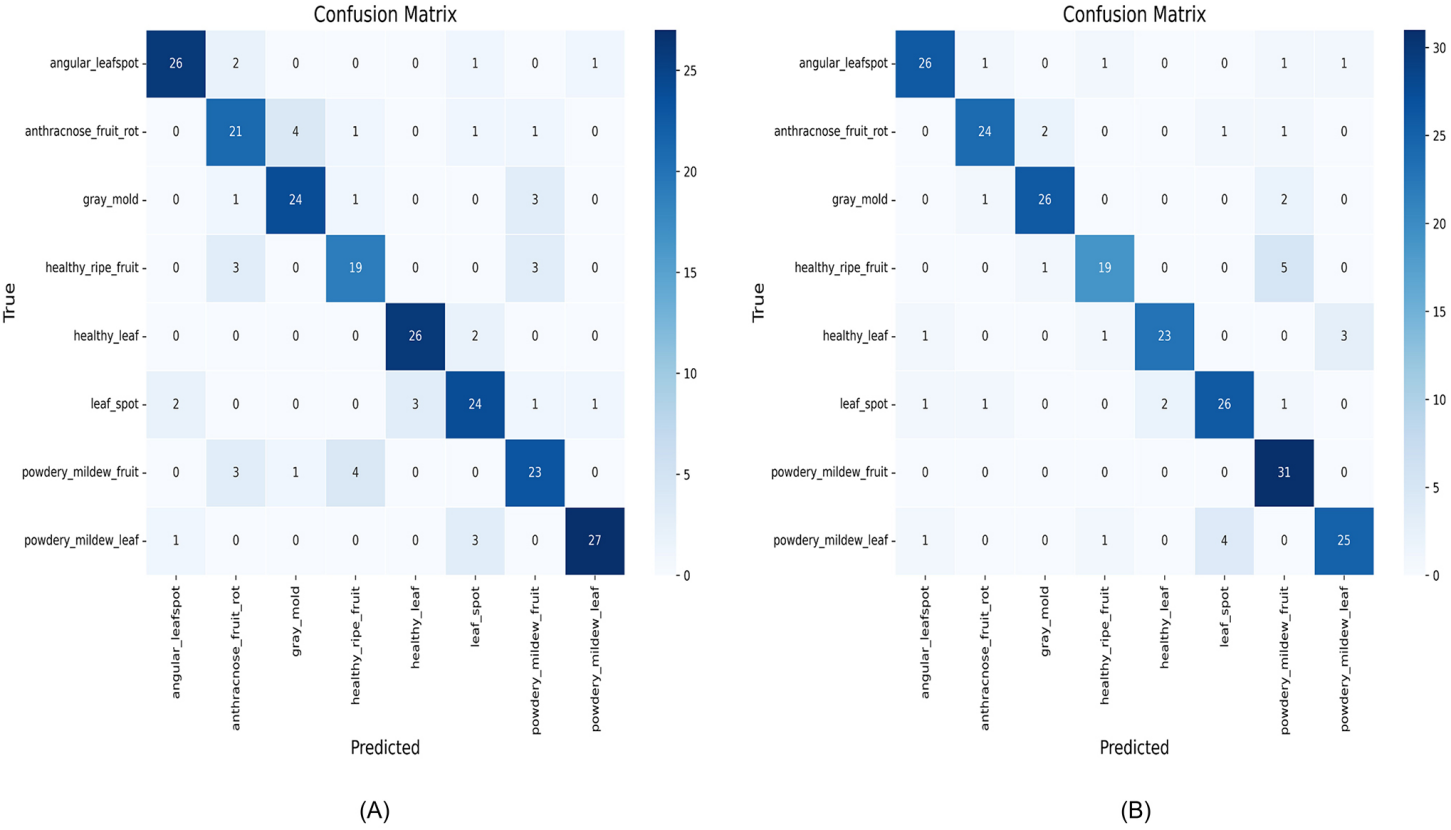
Confusion matrices of the two models before and after the improvement: **(A)** EfficientNetB0; **(B)** ENet-CAEM.

This paper systematically evaluates ENet-CAEM using visual analysis and confusion matrix comparison. **Figure 11** presents validation accuracy trends across epochs for different module combinations, while **Figure 12** depicts the fluctuations in accuracy, precision, recall, and F1 score for the baseline EfficientNetB0 model and each improved model. **Figure 13** shows the curves of performance metrics for the EfficientNetB0 and ENet-CAEM models for different diseases. **Figure 14** employs confusion matrices to provide a detailed comparison of the classification performance of the two models across various categories.

ENet-CAEM demonstrates clear advantages: (1) Higher final accuracy: it outperforms other variants during 150–200 epochs, reflecting enhanced feature extraction and performance on complex data; (2) More stable convergence: its accuracy curve is smoother, indicating improved training stability and reduced noise; (3) Faster early-stage learning: it shows the fastest accuracy growth in the first 50 epochs, suggesting accelerated feature learning.

The ablation experiments confirm progressive performance improvements from Model 1 to Model 6, with ENet-CAEM achieving the best overall results. These findings validate the effectiveness of the proposed architectural and parameter optimizations, providing a reliable technical foundation for practical strawberry disease diagnosis.

An analysis of precision, recall, and F1 score for EfficientNetB0 and ENet-CAEM in strawberry disease recognition shows that ENet-CAEM consistently outperforms EfficientNetB0 across most disease categories. Its higher precision indicates a more accurate distinction between disease samples and background, reducing misclassification. Higher and more stable recall demonstrates a stronger capability to detect diverse disease types, minimizing missed detections. Consequently, the F1 score also remains higher and more consistent, reflecting a balanced and reliable performance across all evaluated categories.

A comparative analysis of the confusion matrices reveals that EfficientNetB0 frequently misclassifies several strawberry disease categories. For instance, angular leafspot, gray mold, and powdery mildew fruit samples are often incorrectly predicted, indicating challenges in distinguishing visually similar or complex diseases. The relatively weak diagonal values reflect limited overall recognition accuracy, with 190 images correctly classified. In contrast, the ENet-CAEM confusion matrix shows clear improvements: the number of correctly classified images increased to 200, a gain of 10 over the baseline, and misclassification rates for anthracnose_fruit_rot, gray mold, leaf spot, and powdery_mildew_fruit were significantly reduced. These results demonstrate that integrating modules such as CCM and MultiScaleECA effectively enhances the model’s recognition of diverse strawberry disease categories.

### 4.7 Explainability analysis

**Figure 15:**
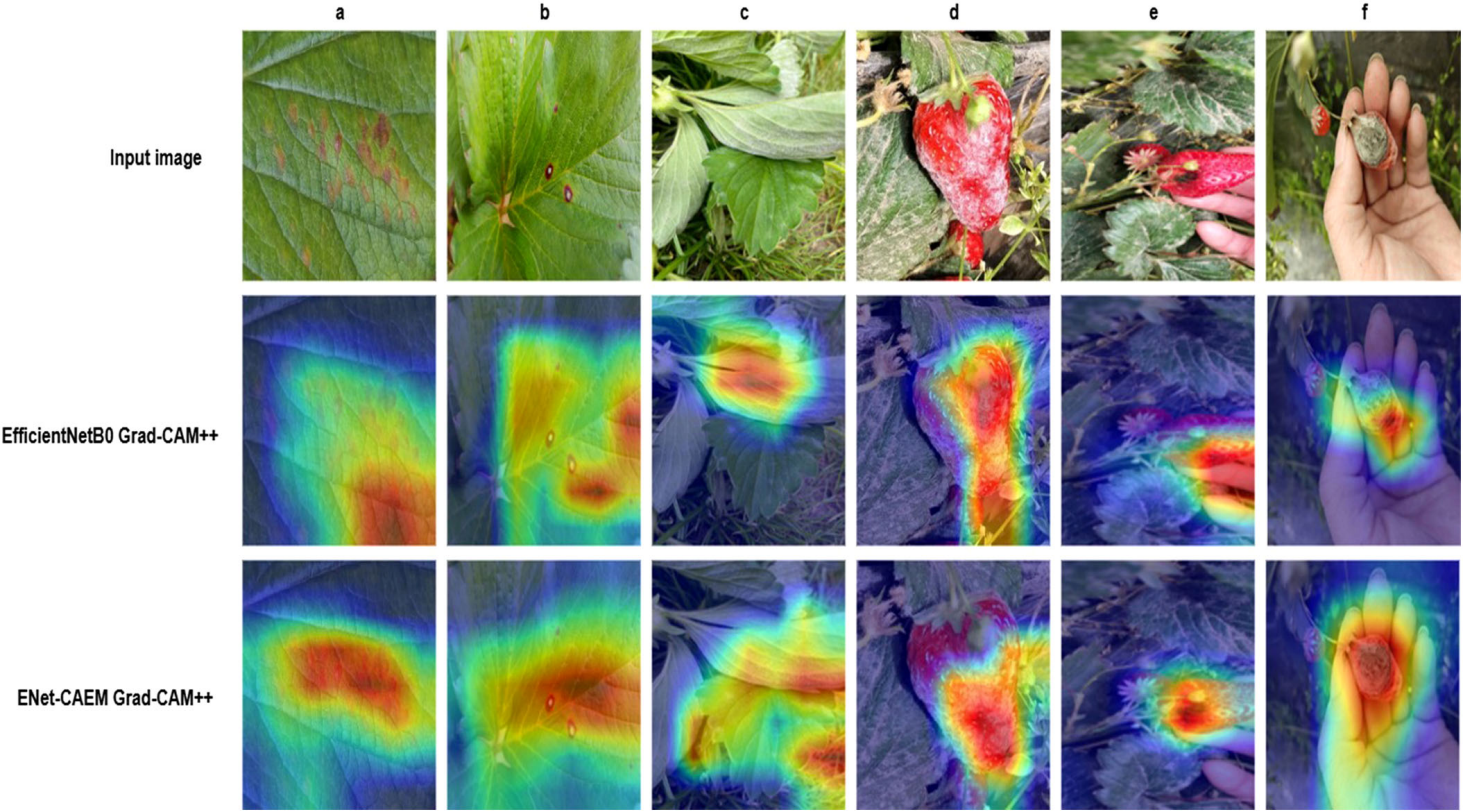
Visualization of the class activation mapping for strawberry disease samples.

To further validate the effectiveness of the improved model, Grad-CAM++ (Chattopadhay et al., 2018) was employed to perform a visual analysis of the model’s discriminative regions. **Figure 15** shows the class activation results for strawberry disease images before and after model refinement, where red areas indicate regions of high attention and blue areas indicate regions of lower attention.

The visualization results reveal that, in some disease samples, the EfficientNetB0 model tends to focus on areas unrelated to lesions while overlooking critical disease information. In contrast, the ENet-CAEM model accurately concentrates on diseased regions with minimal interference from complex backgrounds. Overall, the ENet-CAEM model effectively captures lesion features across different locations and scales, demonstrating superior discriminative power and interpretability.

## 5 Discussion

This study proposes an innovative ENet-CAEM model that systematically addresses key challenges in strawberry disease recognition through the introduction of the CCM, MultiScaleECA, and LightASPP modules.

To evaluate the model’s generalization ability and practical application potential, rigorous cross-domain testing was first conducted on public Kaggle datasets. The experimental results demonstrate that ENet-CAEM maintains stable and excellent recognition performance even when faced with new data differing significantly from the training set distribution. This confirms the model’s strong domain adaptability and cross-scenario robustness, laying a solid foundation for its practical deployment under diverse growth environments and imaging conditions.

Furthermore, a comprehensive evaluation of the ENet-CAEM model was conducted on a self-constructed dataset covering six common strawberry diseases and two healthy states. Compared with the benchmark EfficientNetB0, ENet-CAEM achieved notable improvements across all core metrics, with accuracy, precision, recall, and F1 score increasing by 4.29%, 4.93%, 4.09%, and 4.23%, respectively. Importantly, these gains were achieved while maintaining a competitive parameter count of 6.55 million, highlighting the model’s balanced trade-off between accuracy and efficiency. This balance allows for effective deployment in resource-limited environments.

To further validate the model’s broad applicability, this study compared ENet-CAEM with classical CNN models such as AlexNet, ResNet, and VGG16, as well as recent state-of-the-art models including G-ResNet50, T-CNN, and MS-DNet. The results showed that ENet-CAEM consistently outperformed all competitors across key metrics, demonstrating higher recognition accuracy and greater robustness in handling complex and variable images of leaf and fruit diseases. Moreover, it effectively resolved the performance–efficiency trade-off: ENet-CAEM achieved higher accuracy and F1 scores than the lightweight MS-DNet, while maintaining lower computational complexity and load than models such as T-CNN and G-ResNet50. These findings highlight the model’s superior capability to efficiently extract discriminative features from complex field backgrounds.

In addition, to thoroughly analyze the practical effectiveness of each innovative module, this study conducted detailed ablation experiments. The results clearly demonstrate that modules such as CCM, MultiScaleECA, and LightASPP each made significant contributions to the overall performance improvement of the model. This indicates that the integrated optimization strategy proposed in this study is well-designed and effective, with each component being an indispensable part of achieving high final performance.

In summary, the proposed ENet-CAEM model not only enriches the application of deep learning in agricultural disease identification but also provides an efficient, accurate, and robust technical solution for the intelligent management of the strawberry industry. It holds significant research value and broad prospects for agricultural applications.

## 6 Conclusion

To address the challenges of strawberry disease identification under complex backgrounds, this paper introduces an efficient strawberry disease recognition model, ENet-CAEM, built upon an enhanced EfficientNetB0 architecture. By integrating the CCM, MultiScaleECA, and ASPP modules and incorporating a learnable DropPath regularization mechanism along with a mixed pooling strategy, the model effectively enhances recognition accuracy while maintaining control over parameter size and computational complexity. The experiment was conducted using a self-built dataset comprising images of strawberry diseases, covering six common diseases and two healthy classes. The results demonstrate that ENet-CAEM significantly outperforms the baseline model in accuracy, precision, recall, and F1 score, highlighting its superior recognition capabilities and practicality.

Although ENet-CAEM has demonstrated strong performance in strawberry disease recognition, there is still room for expansion in terms of data diversity and multimodal intelligent modeling. Future research will proceed in two directions:

1. Constructing a more diverse and high-quality strawberry disease image dataset to enhance the model’s ability to recognize different varieties, growth stages, and disease types.
2. Exploring the integration of multimodal fusion technologies and conducting disease progression trend modeling based on temporal image sequences, thereby improving the foresight and decision-support capabilities of strawberry disease recognition.

## Data Availability

The raw data supporting the conclusions of this article will be made available by the authors, without undue reservation.
